# Current strategies for the targeted treatment of high-grade serous epithelial ovarian cancer and relevance of *BRCA* mutational status

**DOI:** 10.1186/s13048-019-0484-6

**Published:** 2019-01-28

**Authors:** Angiolo Gadducci, Valentina Guarneri, Fedro Alessandro Peccatori, Graziana Ronzino, Giuseppa Scandurra, Claudio Zamagni, Paolo Zola, Vanda Salutari

**Affiliations:** 1 Department of Experimental and Clinical Medicine, Division of Gynecology and Obstetrics, University of Pisa, Pisa, Italy; 2 Department of Surgery, Oncology and Gastroenterology, University of Padova, Padova, Italy; 3 Gynecologic Oncology Division, European Institute of Oncology, Milan, Italy; 4 Medical Oncology Unit, Vito Fazzi Hospital, Lecce, Italy; 5 Medical Oncology Unit, Cannizzaro Hospital, Catania, Italy; 6 Addarii Medical Oncology Unit, S Orsola-Malpighi Hospital, Bologna, Italy; 7 Department of Surgical Sciences, University of Turin, Turin, Italy; 8 Department of Health of Woman and Child, Gynecologic Oncology Unit, Catholic University of Sacred Heart, Largo Agostino Gemelli 8, 00168 Rome, Italy; 9 Division of Medical Oncology 2, Istituto Oncologico Veneto, Padova, Italy

**Keywords:** Bevacizumab, BRCA, DNA damage repair, Olaparib, Ovarian cancer, PARP inhibitor, Synthetic lethality

## Abstract

Epithelial ovarian cancer is the most lethal gynecologic malignancy. In most women, it is diagnosed at an advanced stage, which largely explains the poor prognosis of this malignancy. Germline mutations of the genes *BRCA1* and *BRCA2*, which encode proteins essential for the repair of double-strand DNA breaks through homologous recombination, lead to increased cancer predisposition. *BRCA* mutations are present in approximately 14% of epithelial ovarian cancers. Somatic *BRCA* mutations have also been described. Current first-line treatment of high-grade epithelial ovarian cancer includes debulking surgery followed by combination chemotherapy, usually carboplatin and paclitaxel. Ovarian cancer is highly sensitive to chemotherapy, in particular to platinum drugs. Most patient will achieve remission with initial chemotherapy, but most will eventually experience disease recurrence. Targeted therapies, including the anti-angiogenic agent bevacizumab and oral poly (ADP-ribose) polymerase (PARP) inhibitors, have been recently approved for the treatment of ovarian cancer, based on the results from randomized clinical trials showing significant benefits in terms of progression-free survival, with acceptable tolerability and no detrimental effects on quality of life. Olaparib, the first PARP inhibitor to be granted approval, is currently indicated as maintenance monotherapy in ovarian cancer patients with relapsed disease and mutated *BRCA* who have achieved a complete or partial response to platinum-based chemotherapy. The analysis of *BRCA* mutational status has, therefore, also become crucial for therapeutic decisions. Such advances are making personalized treatment of ovarian cancer feasible. Here we briefly review treatments for platinum-sensitive, high-grade serous epithelial ovarian cancer that are currently available in Italy, with a focus on targeted therapies and the relevance of *BRCA* mutational analysis. Based on the evidence and on current guidelines, we propose strategies for the tailored treatment of patients with relapsed ovarian cancer that take into account *BRCA* mutational status and the treatment received in the first-line setting.

## Introduction

Epithelial ovarian cancer is the most lethal gynecologic malignancy [1]. In most patients, ovarian cancer is diagnosed when the disease has progressed to an advanced stage, corresponding to stages IIb to IV of the International Federation of Gynecology and Obstetrics (FIGO) classification, with the involvement of the peritoneal cavity and other organs [2]. This largely explains the poor prognosis of this malignancy.

Epithelial ovarian cancer, which accounts for 90% of primary ovarian tumors, is a heterogeneous disease comprising several histologic subtypes: serous, mucinous, endometrioid, clear cell, transitional cell (Brenner tumors), mixed, and undifferentiated type [2]. The carcinogenesis of epithelial ovarian cancer is not fully elucidated. According to a dualistic model of carcinogenesis, ovarian cancer can be divided into two broad categories, type I and type II [3]. Type I tumors include low-grade serous, mucinous, endometrioid and clear cell carcinomas, and Brenner tumors; they are generally indolent and characterized by mutations of genes involved in signaling pathways (*KRAS*, *BRAF*, *PTEN*, *PIK3CA*, *CTNNB1*, *ARID1A*, and *PPP2R1A*) [3]. Type II tumors are the most prevalent category and include high-grade serous, endometrioid, and undifferentiated carcinomas; they are aggressive, genetically highly unstable, and are usually diagnosed at an advanced stage [3]. Type II tumors rarely harbor the mutations detected in type I tumors, while mutations of *p53* and *BRCA* are common [3, 4]. Mutations of the genes *BRCA1* and *BRCA2* lead to increased cancer predisposition and are present in approximately 14% of epithelial ovarian cancers, according to recent population-based studies [5]. *BRCA1* and *BRCA2* encode proteins that play an essential role in the repair of double-strand DNA breaks through homologous recombination. Somatic *BRCA* mutations and epigenetic inactivation of these genes have been implicated in sporadic ovarian cancer [6, 7].

Low-grade epithelial ovarian cancer, with disease confined to the ovaries and pelvis (FIGO stages I-IIa), is treated with surgical resection (debulking surgery) [8]. In 70% of the cases, this intervention is curative, while 30% are at risk of recurrence [8]. Current first-line treatment of high-grade epithelial ovarian cancer (FIGO stages IIb-IV) includes debulking surgery followed by combination chemotherapy, usually carboplatin and paclitaxel [8]. Ovarian cancer is highly sensitive to chemotherapy drugs, in particular to platinum. While most patient will achieve remission with initial chemotherapy, most will eventually experience disease recurrence [2, 9]. Chemotherapy for relapsed high-grade ovarian cancer includes platinum-based combination regimens for patients with disease recurrence more than 6–12 months after the completion of first-line chemotherapy, and sequential single cytotoxic agents for those with disease recurrence earlier than 6 months after completion of initial chemotherapy [2].

The treatment armamentarium has been recently expanded by the addition of targeted therapies, including bevacizumab, a humanized monoclonal antibody against vascular endothelial growth factor (VEGF), and oral inhibitors of poly (ADP-ribose) polymerase (PARP). With regard to epithelial ovarian cancer, bevacizumab is licensed: i) in combination with carboplatin-paclitaxel, for the front-line treatment of stage IIIB, IIIC and IV cancer; ii) in combination with carboplatin-gemcitabine, for the treatment of the first recurrence of platinum-sensitive cancer not previously treated with anti-angiogenic therapies; iii) in combination with paclitaxel, topotecan, or pegylated liposomal doxorubicin (PLD), for the treatment of platinum-resistant relapsed cancer, after no more than two prior chemotherapy regimens, and not previously treated with anti-angiogenic therapies [10]. Olaparib, the first PARP inhibitor to be granted marketing authorization (in 2014), is licensed in the European Union (EU) as monotherapy for the maintenance treatment of patients with platinum-sensitive relapsed *BRCA*-mutated (germline and/or somatic) high-grade serous epithelial ovarian cancer who are in complete or partial response to platinum-based chemotherapy [11].

The introduction of targeted drugs has significantly increased treatment options and contributed to the development of individualized strategies. *BRCA* mutational analysis has become essential for making therapeutic decisions. In this review, we discuss first- and second-line treatment options currently available in Italy for high-grade serous epithelial ovarian cancer, with a focus on the most relevant findings concerning targeted therapies. We also briefly review the main data highlighting the importance of *BRCA* mutational analysis in the management of patients with ovarian cancer. Based on the reviewed evidence and on current guidelines we propose treatment algorithms for patients with relapsing high-grade, platinum-sensitive ovarian cancer that take into account *BRCA* mutational status and previous exposure to targeted therapies.

## Treatment of high-grade serous epithelial ovarian cancer

### Surgery

Debulking or cytoreductive surgery has a double role in the management of high-grade ovarian cancer because it is not only used for diagnosis and staging, but also as a therapeutic intervention [2]. The goal of primary debulking surgery is to remove all visible disease. The amount of residual disease is an independent prognostic factor of survival, and the absence of macroscopic residual disease is associated with a significantly lower risk of recurrence [8]. Patients not eligible for debulking surgery may benefit from neoadjuvant chemotherapy [12]. Preliminary data from a phase III trial suggest that surgery can be repeated with benefits in highly selected patients with platinum-sensitive disease: in the AGO DESKTOP III/ENGOT ov20 trial, secondary cytoreductive surgery was associated with a clinically meaningful 5.6-month increase of progression-free survival (PFS) [13].

Evidence for the role of hyperthermic intraperitoneal chemotherapy (HIPEC) after cytoreductive surgery upfront are limited. After interval debulking surgery and in the recurrent setting, in a phase III trial that included 245 women who had at least stable disease after three cycles of neoadjuvant chemotherapy with carboplatin plus paclitaxel, the patients who underwent cytoreductive surgery with HIPEC experienced a significantly longer recurrence-free survival (hazard ratio [HR] 0.66; 95% CI, 0.50–0.87) and overall survival (OS) (HR: 0.67; 95% CI, 0.48–0.94) compared to those who underwent cytoreductive surgery alone [14]. The rate of severe adverse events was similar in the two groups. In this context, HIPEC should be performed in clinical trials or in referral centers with high experience in ovarian cancer management.

### First-line chemotherapy

The combination of carboplatin area-under-the-curve (AUC) 5 and paclitaxel (175 mg/m^2^ intravenously over 3 h, every 21 days) remains the standard approach in the first-line setting, despite disappointing results from the long-term follow-up of the registration studies showing relapse rates of 70–80% within the first 2 years [8]. Alternatives to this approach have been extensively studied over the past two decades, but no chemotherapeutic regimen has been conclusively demonstrated as superior to the standard carboplatin-paclitaxel combination [8, 15–18]. Acceptable alternatives include weekly paclitaxel plus every-3-week carboplatin, the addition of bevacizumab to 3-weekly carboplatin-paclitaxel, and intraperitoneal therapy [8, 16, 17].

The recent results of the SOLO-1 trial could define a new standard in first line treatment for women diagnosed with advanced ovarian cancer who carry a *BRCA* 1/2 mutation. SOLO-1 is the first, double-blind, randomised, prospective phase III trial evaluating front line olaparib maintenance therapy after platinum-based chemotherapy in newly diagnosed advanced ovarian cancer (FIGO stage III–IV) with a *BRCA* mutation [18]. A total of 391 patients with high grade serous or endometrioid ovarian cancer who were in clinical complete or partial response after chemotherapy upon entering the study were randomised 2:1 to olaparib tablets 300 mg bd (*n* = 260) or placebo (*n* = 131) for two years. The primary endpoint was investigator-assessed PFS from randomisation. Secondary outcomes included PFS2 (time from randomisation to the second progression), OS and quality of life. Median follow-up was 41 months. PFS2 remained significantly improved among patients who had received olaparib maintenance, with a median PFS2 of 41.9 months for placebo versus median not reached for the olaparib group (HR: 0.50; 95% CI, 0.35–0.72; *P* = 0.0002). There was no clinically relevant change in quality of life between groups and dosing was well tolerated, with only 12% of patients discontinuing olaparib, due to toxicity and not disease progression [18].

### Second-line chemotherapy

Treatment of relapsing ovarian cancer is curative only in a minority of patients. The goals of second-line treatment are to prolong survival, to postpone symptomatic disease progression, and to improve quality of life. Serous histotype, the presence of *BRCA* mutations, tumor size, and the number of metastases are independent predictive factors of response to second-line chemotherapy. A crucial issue in relapsing patients is when to initiate second-line treatment. Evidence suggests that early second-line treatment initiation, prompted by biochemical relapse (i.e., increased level of cancer antigen [CA] 125), is not beneficial [19].

Various options for second-line treatment of relapsed ovarian cancer are available. Treatment choice has traditionally been guided by the sensitivity to platinum-based therapy. Patients sensitive or partially sensitive to platinum, defined respectively by a platinum-free-interval (PFI) > 12 or by a PFI of 6–12 months, are treated with combination chemotherapy, usually platinum-based [8]. A non-platinum option – trabectedin plus PLD – has obtained good results in terms of PFS and OS, and the phase III INOVATYON trial (NCT01379989) is currently comparing this regimen versus the combination of carboplatin plus PLD in this setting [20]. Few second-line options are available for patients resistant to platinum, but the introduction of targeted therapies may improve outcomes also in this difficult-to-treat subgroup.

### Targeted therapies

#### Anti-angiogenic agents

Bevacizumab in combination with chemotherapy has been extensively investigated in various settings of ovarian cancer treatment, including first-line treatment (GOG-0218, ICON7 studies) [21, 22], and treatment of recurrent ovarian cancer in platinum-sensitive patients (OCEANS study) [23, 24], and in platinum-resistant patients (AURELIA study) [25]. Overall, the addition of bevacizumab to chemotherapy has been shown to prolong PFS, with an acceptable tolerability profile and preserved quality of life. In the GOG-0218 study, for example, 1873 women with stage III or IV epithelial ovarian cancer who had undergone debulking surgery were randomized to one of three treatments [21]. All three treatments included 6 cycles of standard front-line chemotherapy (carboplatin-paclitaxel). The control treatment was chemotherapy plus placebo added in cycles 2 through 22; bevacizumab-initiation treatment was chemotherapy plus bevacizumab (15 mg per kg of body weight, every 3 weeks) added in cycles 2 through 6 and placebo added in cycles 7 to 22; bevacizumab-throughout treatment was chemotherapy plus bevacizumab added in cycles 2 through 22. The median PFS was 10.3 months in the control group, compared with 11.2 months in the bevacizumab-initiation group and 14.1 months in the bevacizumab-throughout group. Relative to the control treatment, the hazard ratio for progression or death was 0.717 (95% CI, 0.625–0.824; *P* < 0.001) with bevacizumab-throughout. The difference in PFS between the control group and the bevacizumab-initiation group was not significant, which implies that bevacizumab treatment must be continued beyond chemotherapy to delay disease progression. There was no significant difference in OS among the three groups. The addition of bevacizumab was associated with more adverse events (hypertension and gastrointestinal toxicity), but the rates of gastrointestinal events remained below 3%. No decline in quality of life was reported.

In the OCEANS study that included 484 patients with platinum-sensitive relapsed epithelial ovarian, primary peritoneal, or fallopian tube cancer, median PFS was 12.4 months with bevacizumab (15 mg/kg) added to carboplatin-gemcitabine and 8.4 months in the group treated with chemotherapy alone (HR: 0.484; 95% CI, 0.388–0.605; *P* < 0.001) [23]. Results from the final OS analysis showed no significant difference in OS between patients treated with carboplatin-gemcitabine plus bevacizumab (median OS, 33.6 months) and those treated with chemotherapy alone (32.9 months) [24]. Median follow-up was 58.2 months in the bevacizumab group and 56.4 months in the placebo group. No unexpected safety issues were reported following prolonged exposure to bevacizumab.

The guidelines for ovarian cancer treatment revised in 2017 by the Italian Association of Medical Oncology (AIOM) recommend considering six cycles of bevacizumab in combination with carboplatin-paclitaxel, followed by maintenance monotherapy for the first-line treatment of women with high-grade ovarian carcinoma after both optimal (weak recommendation) and non-optimal (strong recommendation) debulking surgery [8]. In the second-line setting, bevacizumab can be considered in those patients who have not been previously treated with it [8].

#### PARP inhibitors

Repair of DNA damage is essential for the maintenance of genomic integrity. The proteins encoded by the *BRCA1* and *BRCA2* genes are involved in the repair of double-strand DNA breaks. The loss of function of these genes, commonly associated with ovarian cancer, makes cancer cells more dependent on alternative DNA repair processes such as single-strand DNA repair. PARP is an essential component of single-strand DNA repair, and its inhibition prevents cancer cells with deficient *BRCA* function from repairing chemotherapy-induced DNA damage, making them more vulnerable to cytotoxic agents, a concept known in oncology as synthetic lethality [26, 27].

The efficacy of olaparib as maintenance therapy has been demonstrated in randomized, double-blind, placebo-controlled, phase II (Study 19) and phase III (SOLO 2/ENGOT-Ov21) trials [28–30]. In Study 19, conducted in 265 patients with platinum-sensitive, relapsed, high-grade serous ovarian cancer, monotherapy with olaparib 400 mg twice daily (oral capsule formulation) was associated with a significantly longer median PFS compared with placebo (8.4 months versus 4.8 months; HR for progression or death, 0.35; 95% CI, 0.25–0.49; *P* < 0.001) [28]. No significant difference between groups was seen in OS. Adverse events more frequently reported in the group treated with olaparib included nausea, fatigue, vomiting, and anemia, which were mostly of grade 1 or 2. A preplanned analysis of Study 19 data by *BRCA* mutation status showed that patients with platinum-sensitive relapsed serous ovarian cancer with a *BRCA* mutation were more likely to benefit from treatment with olaparib [29]. In the *BRCA*-mutated group, median PFS was 11.2 months in patients treated with olaparib and 4.3 months in those receiving placebo (HR: 0.18; 95% CI, 0.10–0.31; *P* < 0.0001). Median time to first subsequent therapy or death (TFST) and median time to second subsequent therapy or death (TSST) were also analyzed and were, respectively, 15.6 months (olaparib) versus 6.2 months (placebo) (HR: 0.33; 95% CI, 0.22–0.50; *P* < 0.0001), and 23.8 months versus 15.2 months (HR: 0.44; 95% CI, 0.29–0.67; *P* = 0.00013) in patients with a *BRCA* mutation. A final OS analysis following the death of 203 (77%) of the 265 patients in Study 19, after more than 5 years of follow-up, revealed a longer OS of *BRCA*-mutated patients receiving olaparib maintenance therapy, but the differences between groups did not reach statistical significance [30]. The long-term exposure to olaparib was not associated with unexpected safety reports. The efficacy of olaparib (300 mg, twice daily, tablet formulation) as maintenance therapy has been further confirmed in the SOLO 2/ENGOT-Ov21 trial including 295 patients with platinum-sensitive, relapsed *BRCA*-mutated ovarian cancer, who had received at least two lines of previous chemotherapy [31]. Based on the data from the Study 19 and SOLO 2/ENGOT-Ov21 trial, the 2017 AIOM Italian guidelines for the treatment of ovarian carcinoma state that olaparib can be considered following chemotherapy as maintenance therapy in women with *BRCA* mutations [8].

Two additional PARP inhibitors will be available soon: niraparib, approved by the European Medicines Agency (EMA) in November 2017, and rucaparib (EMA approval procedure is ongoing). Niraparib was evaluated in the phase III ENGOT-OV16/NOVA trial in 553 women with platinum-sensitive, recurrent ovarian cancer and was shown to improve PFS substantially and significantly versus placebo, regardless of the presence or absence of germline *BRCA* mutations or homologous recombination deficiency (HRD)-status, extending the potential of PARP inhibition beyond *BRCA*-mutated cancers [32]. Rucaparib was also evaluated in trials in which patients were categorized according to the presence or absence of *BRCA* mutations and to HRD-status [33, 34]. In the phase III ARIEL 3 trial, rucaparib significantly improved PFS over placebo in ovarian cancer patients who had achieved a response to platinum-based chemotherapy, regardless of *BRCA* mutational status or HRD status [33]. Overall, these findings provide further support to the potential of PARP inhibition in the maintenance setting. With regard to the selection of the most appropriate therapy, diagnostic companion testing and resource availability will likely play a central role.

## Analysis of *BRCA* mutational status

Germline *BRCA* mutational status evaluation is recommended for all women diagnosed with nonmucinous, non-borderline, epithelial ovarian, fallopian tube, or primary peritoneal cancer [35]. The goals of genetic testing include completing the diagnostic procedure, guiding therapeutic decisions, and making prevention possible in first-grade relatives of ovarian cancer patients [35]. A positive test in unaffected female relatives may indeed lead to more accurate and frequent monitoring, prophylactic mastectomy, and prophylactic salpingo-oophorectomy with well-established benefits in terms of reduced risk of incident ovarian, fallopian tube and peritoneal cancer [36]. A positive genetic test has important implications also for affected patients because the prognosis is significantly more favorable in patients with mutated *BRCA* (m-*BRCA*), and the presence of *BRCA* mutations is predictive of drug sensitivity to therapeutic combinations with platinum and to PARP inhibitors [36]. Validated procedures for germline mutation testing in peripheral blood are available, while the identification of somatic *BRCA* mutations in tumors is more complex and still suboptimal [37].

Data from the Cancer Genome Atlas (TCGA) have demonstrated that approximately 50% of high-grade serous ovarian cancers are characterized by HRD and suggested that the homologous recombination pathway for DNA repair is not only disrupted by germline and somatic *BRCA* mutations but also by mutations in other genes [38, 39]. This trait of altered DNA repair mechanisms is also known in the literature as “BRCAness,” to indicate the similarity with traits occurring in cancers harboring *BRCA* mutations. Validated procedures for testing HRD and identifying other mutated genes are needed to further select subgroups of patients likely to benefit from novel treatments. Notably, the ARIEL2 study with rucaparib has evaluated the ability of tumor genomic loss of heterogeneity (LOH) to predict response to PARP inhibition, based on the hypothesis that, along with *BRCA* mutations, LOH may represent HRD [34]. The results suggest that LOH may be used to identify patients with wild-type *BRCA* (wt-*BRCA*) who can benefit from treatment with a PARP inhibitor [34].

## Algorithm for the treatment of platinum-sensitive relapsed epithelial ovarian cancer

**Fig. 1:**
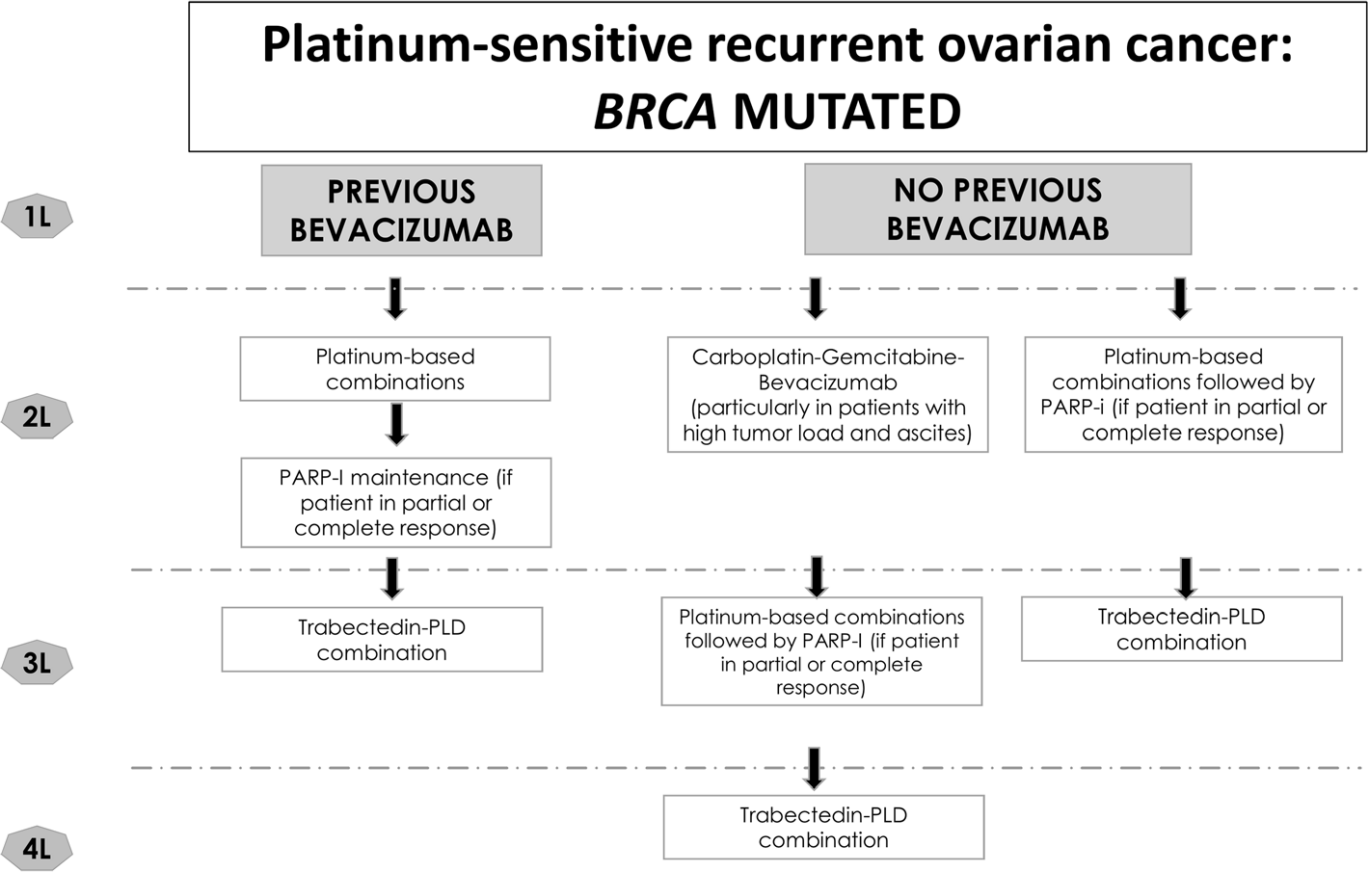
Algorithm for the treatment of patients with recurrent platinum-sensitive, epithelial ovarian cancer and m-*BRCA*. PARP-I, PARP inhibitor; PLD, pegylated liposomal doxorubicin

**Fig. 2:**
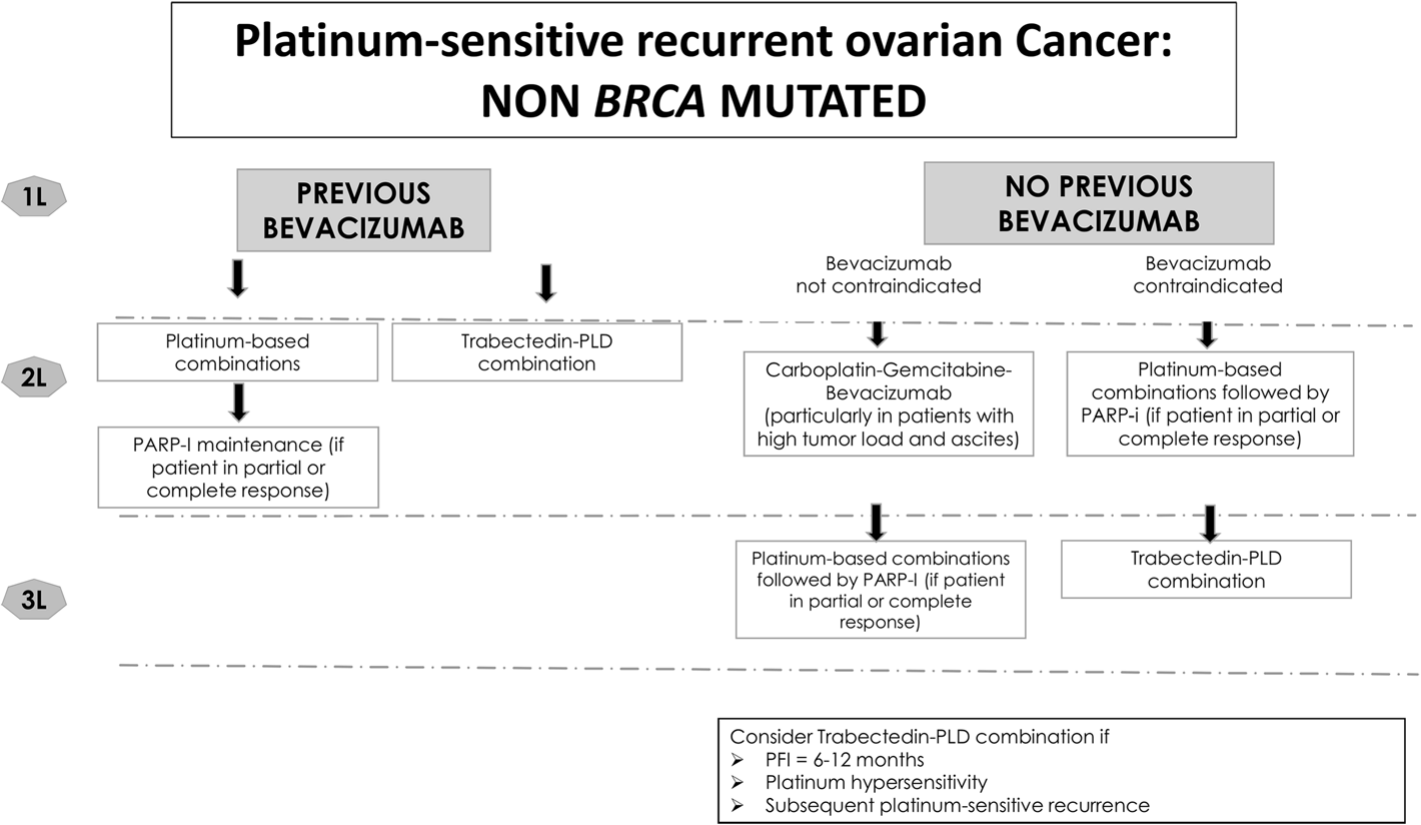
Algorithm for the treatment of patients with recurrent platinum-sensitive, epithelial ovarian cancer and wt-*BRCA*. PARP-I, PARP inhibitor; PLD, pegylated liposomal doxorubicin; PFI, platinum-free interval

The strategy we suggest for the treatment of patients with relapsed, platinum-sensitive ovarian cancer is tailored to patients based on whether they have been treated with bevacizumab in the first-line setting and on their *BRCA* mutational status (Figs. 1 and 2).

In our clinical practice bevacizumab is generally used in combination with carboplatin-paclitaxel in the first-line setting, unless contraindicated. Typical contraindications for the use of bevacizumab are the complications associated with major abdominal surgical procedures. As recommended by current guidelines, bevacizumab can be used regardless of the presence of residual disease following debulking surgery [8]. The evidence supporting this approach is, however, stronger in patients with suboptimal debulking surgery [8]. Further evidence about the efficacy and safety of bevacizumab in this setting will hopefully be provided by further research, including the ongoing trial MITO16-MANGO2 (NCT01706120). This trial is investigating potential clinical and biological prognostic factors in ovarian cancer patients receiving a combination of bevacizumab and carboplatin-paclitaxel as first-line treatment.

Decisions concerning maintenance therapy in platinum-sensitive disease are currently made based on *BRCA* mutational status. *BRCA* mutational status is the first defined predictive marker guiding therapeutic decisions in ovarian cancer, In our practice, olaparib is the targeted therapy of choice for maintenance treatment in patients with m-*BRCA* who have achieved a partial or complete response to second-line platinum-based chemotherapy (Fig. 1) [11]. *BRCA*-mutated patients not previously treated with bevacizumab, and with no contraindications to anti-angiogenic therapy, can be offered the option of treatment with bevacizumab in the second-line setting, before considering olaparib (Fig. 1). According to the results from the OCEANS trial, patients with very high disease burden (e.g., patients presenting with ascites and/or pleural effusion), who have not received bevacizumab in combination with chemotherapy as first-line, may still benefit from the addition of bevacizumab to second-line chemotherapy [23, 24].

In patients with platinum-sensitive relapsed ovarian cancer with wt-*BRCA*, treatment decisions for the second-line setting also depend on prior exposure to bevacizumab (Fig. 2). Second-line options include platinum-based combination chemotherapy for those patients who have already been treated with bevacizumab in the first-line setting. For patients not previously treated with bevacizumab, second-line options include the addition of bevacizumab to the combination carboplatin-gemcitabine according to the EMA indication, or a platinum-based combination without the addition of bevacizumab if this agent is contraindicated [10].

## Conclusions and future perspectives

The recent addition of targeted therapies – anti-angiogenic agents and PARP inhibitors – to the pharmacologic treatments available for ovarian cancer has improved patient outcomes while increasing the available options for the traditionally difficult-to-treat disease. The correct sequence of treatments, including the decision of adding bevacizumab to the first- or second-line treatment, is mainly based on clinical factors. The analysis of *BRCA* mutational status has allowed the first step into individualized strategies for the management of patients with ovarian cancer. This analysis remains crucial and should be offered routinely at the time of diagnosis to all patients. The development of new PARP inhibitors, which also have proven effective in patients with wt-*BRCA*, and the advances in our understanding of HRD will further improve patient selection and extend the usefulness of targeted therapies for ovarian cancer. In this respect, ongoing trials of particular interest are those investigating PARP inhibitors in patients with mutations in homologous recombination repair-associated genes (e.g., NCT02354131 and NCT02655016). Other interesting ongoing lines of research are addressing the combination of targeted therapies (e.g., anti-angiogenic agents with PARP inhibitors) [40, 41]. We also need to understand the long-term tolerability of the various PARP inhibitors, whether they have distinct safety profiles, and the mechanisms leading to the development of resistance. Future studies should focus on the optimization of tolerability and the assessment of patient-reported outcomes including quality of life to improve our understanding of the effects of prolonged maintenance treatment with targeted therapies compared to those of intermittent chemotherapy. Finally, considering the results of SOLO-1 trial, future studies should clarify the role of PARP inhibitors maintenance after previous PARP inhibitors exposure.

## Abbreviations

AIOM: Italian Association of Medical Oncology
CA: cancer antigen
EMA: European Medicines Agency
EU: European Union
FIGO: International Federation of Gynecology and Obstetrics
HRD: homologous recombination deficiency
LOH: loss of heterogeneity
OS: overall survival
PARP: Poly (ADP-ribose) polymerase
PFI: Platinum-free interval
PFS: Progression-free survival
PLD: Pegylated liposomal doxorubicin
TCGA: The Cancer Genome Atlas
TFST: Time to first subsequent therapy or death
TSST: Time to second subsequent therapy or death
VEGF: Vascular endothelial growth factor
